# Post-partum Hospital Stay and Mothers' Choices on Breastfeeding and Vaccines: A Chance We Should Not Miss

**DOI:** 10.3389/fpubh.2021.625779

**Published:** 2021-05-28

**Authors:** Daniela Morniroli, Alessandra Consales, Luana Riverso, Lorenzo Colombo, Elena Nicoletta Bezze, Patrizio Sannino, Lidia Zanotta, Paola Marchisio, Fabio Mosca, Laura Plevani, Maria Lorella Giannì

**Affiliations:** ^1^Fondazione IRCCS Ca' Granda Ospedale Maggiore Policlinico, NICU, Milan, Italy; ^2^Department of Clinical Sciences and Community Health, University of Milan, Milan, Italy; ^3^Direzione Professioni Sanitarie, Fondazione IRCCS Ca' Granda Ospedale Maggiore Policlinico, Milan, Italy; ^4^Fondazione IRCCS Ca' Granda Ospedale Maggiore Policlinico, Pediatric Highly Intensive Care Unit, Milan, Italy; ^5^Department of Pathophysiology and Transplantation, University of Milan, Milan, Italy

**Keywords:** breast milk, breastfeeding, vaccinations, maternal education, knowledge, healthcare professionals, post-partum

## Abstract

Parents' education and knowledge regarding major topics of children's health, such as nutrition and vaccines, have a paramount role. However, breastfeeding rates in first year of life are lower than recommended, and vaccine hesitancy is progressively spreading. To reverse this harmful trend, healthcare professionals are challenged to promote correct health information. This study aimed to assess newly mothers' knowledge of breastfeeding and vaccinations, and education received on both topics during hospital stay. We performed a cross-sectional survey in the Postnatal Unit of our Center. Mothers of full-term babies with a birthweight >2,500 g were enrolled. Two different questionnaires, one about breastfeeding and one about vaccines, were proposed to the 140 enrolled mothers. Ninety-nine percent of mothers enrolled were aware of breastfeeding benefits, and 92% felt adequately supported by maternity staff. Less than 25% stated to have received sufficient information regarding breastfeeding. Only 20% of mothers received information about vaccines during hospital stay. Healthcare providers were identified as primary, secondary, and tertiary source of information on vaccines by 55, 15, and 30% of mothers, respectively. Healthcare professionals are crucial in informing and educating mothers on breastfeeding and vaccinations. Post-partum hospital stay could be the right time for this critical responsibility.

## Introduction

Increasing evidence indicates that investments through the first 1,000 days of life, continuing up to adolescence, play a key role in the optimization of health and development outcomes across the lifespan. Among the proposed interventions, the importance of improving parents' knowledge on major topics of children's health, such as nutrition and prevention of infectious diseases through vaccines, has been highlighted (1). Indeed, both the promotion of exclusive breastfeeding and vaccinations are commonly recognized as two of the most cost-effective public health interventions currently available (2, 3). In this context, breastfeeding promotion and support immediately after birth are critical for its successful initiation and duration (2). In light of the important role of breastfeeding in complementing vaccinations, the association of breastfeeding promotion and support with vaccination counseling has also been suggested (4). Newly mothers have been reported to think and search for information regarding childhood vaccines already in the early post-partum period confirming that maternity health care is an important platform for health promotion (5, 6).

It is already well-known how the first years of life represent a window of opportunity for the establishment of a positive “programming” effect that could influence health in later stages of life (7). In particular, exclusive breastfeeding in the first 6 months of life and its continuation during complementary food introduction has a substantial short and long-term impact on infants' health (8). Likewise, vaccinations, due to their paramount role in protecting children and future adults from diseases that could have lifelong consequences, represent a key public health issue. Surprisingly, in high-income countries, breastfeeding rates in babies' first year of life are lower than recommended (9) and vaccine hesitancy is progressively spreading (10). Indeed, the lack of confidence in vaccines is currently threatening the success of vaccination programs worldwide, decreasing vaccine coverage and increasing the risk of vaccine-preventable disease outbreaks (11). Various controversies and vaccination scares over the years, supported by sometimes excessive and inappropriate media coverage, have affected parents' trust in vaccines (12). Another reason to be considered among the probable causes of vaccine hesitancy is the growth of “healthcare consumerism,” which has prompted greater patients' involvement in their own health decisions (13). Indeed, the rise of the “informed patient/parent” is a double-edged weapon, highly dependent on where such information comes from.

To reverse this harmful trend leading to a decline in exclusive breastfeeding and vaccination rates, healthcare professionals are challenged to take every chance to promote correct health information. Post-partum hospital stay could be an unmissable opportunity to correctly advise mothers on significant health choices for their babies.

The aim of this study was to investigate newly mothers' breastfeeding benefits and vaccines knowledge, breastfeeding support and information received during hospital stay, information received on vaccines, if any, false beliefs on vaccines and source of information.

## Materials and Methods

### Design and Setting

We performed a cross-sectional survey from March 2017 to September 2017. The study was conducted in the Postnatal Unit of Fondazione IRCCS Ca' Granda Ospedale Maggiore Policlinico in Milan, northern Italy. The Clinic is a referral center, admitting pregnant women resident in all Italian regions and covering ~6,000 deliveries per year. The hospital comprises a neonatal Level III center.

The Institutional Ethics Committee approved the study and written informed consent was obtained from all participants.

### Sample

Mothers of newly-born infants discharged from the maternity ward were invited to take part in the survey. We enrolled Italian speaking mothers who had given birth to a full-term baby (born between 37 and 42 weeks of gestational age) with a birthweight >2,500 g. Exclusion criteria were: any contraindication to breastfeeding and/or personal choice not to breastfeed.

### Data Collection

Participation in the research was voluntary and anonymous. Mothers were enrolled at discharge. The survey was proposed by healthcare providers (pediatric nurse or neonatologist) but was self-administered and paper-based, requiring ~15 min to be completed. At enrolment, maternal socio-demographic characteristics and pregnancy duration were collected. Data regarding mode of feeding were obtained from neonatal computerized medical charts (Neocare, i & t Informatica e Tecnologia Srl, Italy) and categorized according to the World Health Organization (WHO), in exclusive, predominant and complementary breastfeeding, and no breastfeeding (14).

### Instruments

Two different questionnaires were designed by field experts (Table 1): one about breastfeeding and one about vaccines. The former was developed by a pediatrician, a neonatologist and a certified lactation consultant who conceived 5 yes-or-no questions on mothers' knowledge of breastfeeding benefits (items 1–2), perception of in-hospital support and information received during hospital stay on the main topics of breastfeeding, as listed in the WHO/UNICEF “Ten Steps to Successful Breastfeeding” (items 3–5). Answers to items 1–3 could be complemented with a brief open statement.

**Table 1:** Questionnaires on breastfeeding and vaccines.

**Part One—breastfeeding**	**Yes**	**No**
1) Are you aware of breastfeeding benefits for mother and child?		
If yes, please indicate which ones you remember.		
2) Did you attend a birth class?		
If yes, were breastfeeding benefits discussed?		
3) Did you feel adequately supported with regard to breastfeeding during your hospital stay?		
If yes, please state why.		
If no, please state why.		
4) Please, indicate which of the following information has been provided to you, during your hospital stay.		
Mother's milk benefits.		
Correct latching and breastfeeding positions.		
Infant's early hunger cues.		
Hand expression techniques.		
Human milk extraction and storage.		
Alternative feeding methods.		
Pacifier use.		
5) Have you received conflicting information about breastfeeding by healthcare professionals?		
**Part two—vaccines**	**Yes**	**No**
1) Have you received information about vaccines?		
2) From whom have you received information about vaccines? (Please rate from 1 to 3)Healthcare Professionals. Friends and relatives. Other sources (TV, Internet, social media, etc.).		
3) Do you know the vaccination timetable?		
4) Are vaccine-preventable diseases no longer a threat or have they been eradicated?		
5) Are vaccines related to autism?		
6) Are vaccines not 100% effective?		
7) Could vaccines weaken children's immune system?		

The second questionnaire was designed by an expert in the field of pediatric immunology and aimed at assessing mothers' knowledge on vaccinations (items 3–7) and the information received on this topic (items 1–2). Item 2 asked mothers to rate primary, secondary and tertiary source of information on vaccinations from 1 to 3. The remaining items were yes-or-no questions.

The questionnaires were preliminarily administered to a sample of 50 Italian speaking mothers to ascertain items' comprehension; these 50 mothers were not considered part of the present study population, nor were they included in the statistical analysis.

### Data Analysis

Data were expressed as mean ± standard deviation (SD) or percentage (%). Associations between basic characteristics of enrolled mothers and answers to the questionnaires were assessed using the chi square test. For statistical analysis purposes, age was categorized according to median value (≤35 and >35 years) and education level was divided into ≤13 years (high school or lower) and >13 years (college degree or higher). A 2-sided *p*-value <0.05 was considered statistically significant. Statistical analysis was performed using SPSS version 25 statistic software package (SPSS Inc., Chicago, IL, USA).

## Results

Of the 171 mothers whom the study was proposed to, 140 mothers were enrolled and completed the questionnaires. Basic characteristics of enrolled women are shown in Table 2.

**Table 2:** Basic characteristics of enrolled mothers.

	**Mean (SD)**
Age (years)	35 (4.10)
Gestational age (weeks)	39 (1.01)
	***N*** **(%)**
Primiparous	73 (52)
Cesarean section	29 (21)
Married	85 (61)
College degree	92 (66)
High-school diploma	139 (99)

*Continuous variables are expressed as mean ± standard deviation (SD), whereas categorical variables as absolute number (N) and percentage (%)*.

At discharge, the majority of mothers were exclusively breastfeeding (93%), while 5% practiced complementary breastfeeding.

### Breastfeeding Questionnaire

The vast majority of mothers were aware of breastfeeding benefits (99%). The benefits most frequently reported were: passive transfer of antibodies and overall improvement of infant's immune system (96%), optimal nutritive content of human milk (68%), improvement of mother-infant bonding (38%), and beneficial effects on mothers and infants' health (38%). Other benefits listed regarded mothers' physical shape and fitness (27%), and economic advantage (32%).

A total of 47% of mothers had attended a birth class. Among them, 94% stated that breastfeeding benefits had been discussed. Primiparas, mothers with an educational level >13 years and age ≤35 years attended birth classes in a higher percentage of cases than multiparas, mothers with an educational level ≤13 years and age >35 years, respectively (Table 3).

**Table 3:** Association between basic characteristics of enrolled mothers and birth class attendance.

	**Prenatal class attendance**	
	**Yes (%)**	**No (%)**
**Education**		
>13 years	53^*^	47
≤13 years	33	67
**Age**		
25–35 years	55^*^	45
36–46 years	37	63
**Parity**		
Primipara	78^**^	22
Multipara	13%	87

*Data are expressed as percentage (%)*.

* *p < 0.05;*

** *p < 0.0001*.

The large majority of women felt adequately supported in breastfeeding by maternity ward staff (92%), listing helpfulness, expertise and kindness as their most valuable qualities. Among those that answered negatively, 35% declared they did not receive breastfeeding support by healthcare professionals during hospital stay, while 28% thought that information provided during hospital stay was insufficient.

Mothers were informed about correct latching and breastfeeding positions in 74% of cases and about infant hunger cues and breastfeeding benefits in 51 and 44% of cases, respectively. Less than 25% of mothers declared to have received information regarding hand expression, milk expression and storage, alternative methods of feeding and pacifier use (Table 4). Approximately 15% of mothers reported having received contrasting information by healthcare providers during hospital stay.

**Table 4:** Answers to item 4 of the breastfeeding questionnaire: information provided by healthcare staff during hospital stay.

**Information provided**	**Yes (%)**	**No (%)**
Mother's milk benefits	44	56
Correct latching and breastfeeding positions	74	26
Infant's early hunger cues	51	49
Hand expression techniques	24	76
Human milk extraction and storage	16	84
Alternative feeding methods	10	90
Pacifier use and limitations	19	81

*Data are expressed as percentage (%)*.

### Vaccination Questionnaire

Only 20% of mothers declared to have received information about vaccines during hospital stay.

Healthcare providers were identified as primary, secondary and tertiary source of information by 55, 15 and 30% of mothers, respectively. Friends and relatives contributed as the first source for 21% of mothers and other sources of information (i.e., internet and social media) for 24% of women, as showed in Table 5.

**Table 5:** Sources of information on vaccines in order of contribution.

	**Healthcare professionals (%)**	**Friends and relatives (%)**	**Other sources (%)**
First	55	21	24
Second	15	53	32
Third	30	26	44

*Data are expressed as percentage (%)*.

First time mothers reported that healthcare professionals were their primary source of vaccine information in a lower percentage of cases, compared to multiparas. Specifically, primiparas reported healthcare professionals as primary source in less than one third of cases whereas friends and “Other sources” (TV, Internet, social media, etc.) were listed in the majority of cases (Table 6).

**Table 6:** Association between parity and primary source of information on vaccines.

	**Source**	**Rated #1 (%)**
Primiparas	Healthcare professionals	28^**^
	Friends/relatives	33
	Other	39
Multiparas	Healthcare professionals	75
	Friends/relatives	12
	Other	13

** *p < 0.0001*.

Half of mothers (51%) reported they knew of the vaccination timetable. Among those, primiparas were less aware of the vaccination timetable than multiparas (18 vs. 82%, *p* < 0.0001). No statistical difference was found for maternal age or education level. Many mothers stated that vaccine-preventable diseases are no longer a threat (60%) and that vaccines are not 100% effective in preventing diseases (57%). Only 10% of women answered that vaccines are related to autism, whereas nearly a quarter of women declared that vaccines could weaken the infant's immune system.

## Discussion

The results of the present study indicate that, although mothers enrolled were aware that breastfeeding is beneficial, they lacked a thorough knowledge of its benefits, which, in turn, could lead to an underestimation of breastfeeding importance for the dyad's health. For the most part, maternal knowledge appears to be limited to the benefits of breastfeeding on a newborn's immune system. Whereas, the nutritional benefits of mother's milk (68%), as well as other beneficial effects that come from close bonding between newborn babies and their mothers (38%) were only reported by a limited percentage of study participants. Moreover, the breastfeeding information received by most of the mothers during their hospital stay focused exclusively on correct latching and breastfeeding positions, which indicates that the mothers did not receive comprehensive information.

With regard to vaccinations, although healthcare workers have a crucial role in informing newly mothers on a wide range of good health practices, in our study only 20% of mothers declared they had received vaccine information during their post-partum hospital stay and nearly half, particularly primiparas, were uninformed about vaccination timetables, at discharge. This gap of information offered by healthcare staff opens the way to other unofficial sources of information, such as relatives, friends and social media, leading to an increased risk of misinformation.

Consistently, in our study, healthcare professionals are indicated as the primary source of vaccine information by only 55% of mothers, with nearly a third rating them as the last source of knowledge. Lack of information during hospital stay combined with sources of information other than healthcare professionals may at least partially explain the maternal misinformation reflected by the answers given to the questionnaires, that in most cases were not supported by scientific evidence.

Despite great efforts by the world public health institutions, parents' appropriate scientific information on breastfeeding and vaccines is still a global challenge. Low rates of breastfeeding and vaccine hesitancy are a major concern within the Italian context, with an increasing tide of vaccine refusal or delay (15) and low breastfeeding continuation rates in the first months after hospital discharge (16). Among determinants of breastfeeding initiation, attending a birth class is a known promoting factor, whose effect can impact breastfeeding rates up to 6 months, as reported in a study by Rosen et al. (17). The present study indicates that only half of the enrolled mothers attended a prenatal class and, among them, the percentage of primiparas was higher than that of multiparas (78 vs. 13%, respectively). In line with this finding, first-time mothers have been reported to be more likely to attend birth classes (18). However, if prenatal education is lacking, in-hospital information and support become even more crucial, as highlighted in an increasing amount of literature (19). In this study, nearly all enrolled mothers declared they had received adequate breastfeeding assistance. This positive finding could be the result of the hospital efforts within the Baby Friendly Hospital Initiative (20) accreditation, which was ongoing during the present study. Despite this result, the breastfeeding information mothers received was not extensive. Nor did it cover important information that could promote breastfeeding after discharge, such as infant's early hunger cues or milk expression and storage techniques. Since overall breastfeeding rates after discharge across Europe are lower than recommended (21), appropriate and timely information about breastfeeding issues after discharge could be key to increasing breastfeeding rates. Moreover, conflicting information by healthcare professionals was reported by 15% of mothers. Although this is a minor percentage, when taking into account how healthcare workers can impact mothers' knowledge, evidence-based information and education should be offered by hospital staff, as highlighted by newly mothers themselves in an exploratory study by Hauck et al. (22).

While the importance of in-hospital breastfeeding support has been thoroughly investigated, educational interventions regarding vaccinations in this critical time period have been poorly explored, while at the same time vaccine hesitancy has become a large-scale health concern (23). Vaccine refusal in Italy has become a major health threat in the last decade, causing small epidemic flares of preventable infectious diseases, such as measles (24). As a consequence, since 2017, unvaccinated children up to 6 years are not allowed to attend nurseries and kindergardens, and parents of unvaccinated older children are fined (25). To understand the underlying reasons of vaccine hesitancy, several studies have been conducted, that also have explored healthcare professionals' knowledge and personal vaccine hesitancy (26).

Surprisingly, although healthcare workers' role is crucial in informing newly mothers about good health practices, in our study, among primiparas, only 18% were informed on vaccination timetables at discharge. Moreover, healthcare professionals were listed as primary source in just 21% of cases. These results indicate that primiparas are more susceptible to misinformation than multiparas and could benefit the most from vaccine information during post-partum hospital stay.

The need for scientific-based information provided by healthcare workers has been underlined in many countries across the world (27). Despite that, the Italian healthcare system has hardly considered in-hospital post-partum stay as an appropriate and timely setting for vaccine education, while a large study conducted in France has demonstrated how post-partum vaccination promotion through motivational interviews is an effective tool for increasing vaccination rates at 7 months of age (28).

The present study has two main limitations. Firstly, it is a monocentric study conducted within a metropolitan city setting so that the results may not be generalizable to other settings. However, our sample may be considered representative of the Italian population, since the population enrolled in this study mirrored data from the Italian National Institute of Statistics, whose annual report on Italy's birth rates shows a mean age at childbearing of 32.5 years with nearly a half of newborns being firstborns (29). Secondly, no follow-up data are available to evaluate the effect of the information received during hospital stay on breastfeeding outcomes and attitudes toward vaccines.

Our study highlights the importance of healthcare professionals' role in informing mothers on these two crucial health topics, and the post-partum hospital stay as an effective time for this important responsibility. Further studies are needed to assess the intervention feasibility and efficacy in promoting children's health.

## Data Availability Statement

The datasets presented in this article are not readily available because, due to the sensitive nature of the questions asked in this study, survey respondents were assured that raw data would remain confidential and would not be shared. Requests to access the datasets should be directed to maria.gianni@unimi.it.

## Ethics Statement

The studies involving human participants were reviewed and approved by the Institutional Ethics Committee of the Fondazione Istituto di Ricovero e Cura a Carattere Scientifico Ca' Granda Ospedale Maggiore Policlinico, Milan. Informed written consent was obtained from the participants before enrolment. The patients/participants provided their written informed consent to participate in this study.

## Author Contributions

MG, EB, LZ, and PS contributed to conception and design of the study. LC, LP, LZ, and MG organized the database. MG, LR, and DM performed the statistical analysis. DM and LR wrote the first draft of the manuscript. MG and AC critically revised and edited the manuscript. LP, PM, and FM supervised the research. All authors contributed to manuscript revision, read, and approved the submitted version.

## Conflict of Interest

The authors declare that the research was conducted in the absence of any commercial or financial relationships that could be construed as a potential conflict of interest.
